# Residual Eosinophilic Inflammation of Sinonasal Mucosa Under the Maintenance Phase of Eosinophilic Granulomatosis With Polyangiitis

**DOI:** 10.7759/cureus.91556

**Published:** 2025-09-03

**Authors:** Masaaki Ishikawa, Kazuma Nishisaka, Mie Inoue, Shizuko Ainai, Goichi Kageyama

**Affiliations:** 1 Department of Otolaryngology, Hyogo Prefectural Amagasaki General Medical Center, Amagasaki, JPN; 2 Department of Rheumatology and Clinical Immunology, Kobe University Graduate School of Medicine, Kobe, JPN; 3 Department of Rheumatology, Hyogo Prefectural Amagasaki General Medical Center, Amagasaki, JPN

**Keywords:** chronic rhinosinusitis, eosinophilia, eosinophilic granulomatosis with polyangiitis, mepolizumab, nasal polyps

## Abstract

Objective: Comorbid chronic rhinosinusitis (CRS) can be observed during the maintenance phase of eosinophilic granulomatosis with polyangiitis (EGPA), particularly during the tapering of oral corticosteroid (OCS) dosages, even when treated with mepolizumab (MPZ). Control of the comorbid CRS might be key for the remission of EGPA with lower OCS dosages, but the clinical features remain unclear. This study retrospectively investigated the clinical features of comorbid CRS during the EGPA maintenance phase.

Methods: 11 patients were included in this study. Clinical data, including details of the EGPA treatment, were analyzed. The histopathological eosinophilic count was assessed to determine the phenotype of the comorbid CRS. To assess the severity, the criteria defining primary eosinophilic CRS (eCRS) were used: a histopathological eosinophilic count ≥10 per high-power field.

Results: The median SNOT-22 score (interquartile range [IQR]) was 23 (15−55), and six (55%) patients were classified into the moderate/severe category of SNOT-22 stratification. Nine (82%) patients had nasal polyps (NPs). Six (55%), seven (64%), six (55%), and two (18%) patients received inhaled corticosteroids, MPZ, immunosuppressants, and nasal corticosteroids, respectively. All patients received OCS, and the median value (IQR) was 4 (1−5) mg/day. In absolute eosinophil count (AEC) of blood tests, one patient (9%) exhibited blood eosinophilia (AEC ≥500/μL), and the median value (IQR) was 52 (29−371) /μL. In the histopathological eosinophilic count of the sinonasal mucosa, 10 (91%) patients met the diagnostic criteria for primary eCRS, with a median value (IQR) of 136 (78−134) cells/high-power field.

Conclusions: NPs with eosinophilic inflammation without blood eosinophilia can be observed during the maintenance phase of EGPA. Current treatments, including MPZ for EGPA and its comorbidities, may be insufficient to control comorbid CRS. Endotypic investigation of the sinonasal mucosa is required to elucidate its pathophysiology.

## Introduction

The European Position Paper on Rhinosinusitis and Nasal Polyps (EPOS 2020) defines the chronic rhinosinusitis (CRS) classification [1]. In primary diffuse CRS, endotype dominance is classified as type 2 (T2) and non-T2. Among phenotypes, CRS with nasal polyps (CRSwNPs) and eosinophilic CRS (eCRS) fall under T2 CRS, whereas non-eCRS is classified as non-T2 CRS. In primary diffuse CRS, eCRS is recognized as a subgroup of CRSwNPs (e.g., eCRSwNPs) [2]. The classification concept is based on a strong association between eosinophils and T2 cytokines [1]. The accumulation of eosinophils in NPs can be controlled by several factors (e.g., IL-4- and IL-13-derived increase of adhesion molecules such as vascular cell adhesion molecule 1 and intercellular adhesion molecule 1; chemokine (C-C motif) ligand 13 and 26 for eosinophil recruitment; IL-5 for eosinophil survival) [3]. Primary diffuse T2 CRS (CRSwNPs/eCRS) can exhibit high relapse/refractoriness [1]. For instance, patients with moderate to severe eCRS exhibit greater postoperative refractoriness than those without eCRS [4]. Although a short course of oral corticosteroid (OCS) and endoscopic sinus surgery is standard treatment, T2 biologics can be used for CRSwNPs (eCRSwNPs) resistant to standard treatments [1,2]. Recent studies have reported effects of T2 biologics on primary diffuse T2 CRS, including anti-interleukin (IL)-4Rα chain antibody (dupilumab), anti-IL-5 antibody (mepolizumab, MPZ), and anti-IL-5 receptor antibody (benralizumab) [2,5]. Responsiveness to biologics varies [6], possibly due to the endotypic heterogeneity of CRS. Primary diffuse CRS endotypes include T1, T2, T3, and mixed patterns [7]. These findings suggest that primary CRS should be classified based on endotypic heterogeneity rather than a simple binomial classification focused solely on T2 inflammation.

Eosinophilic granulomatosis with polyangiitis (EGPA) is a rare systemic disease characterized by the triad of asthma, CRS, peripheral blood eosinophilia, and granulomatosis or vasculitis, affecting multiple organs [8]. The pathophysiology is mainly driven by T2 cytokines, particularly IL-5 [8]. OCS is a representative treatment modality for EGPA. In the maintenance phase of EGPA, T2 biologics (e.g., MPZ) allow clinicians to achieve EGPA remission with lower OCS doses, such as a Birmingham Vasculitis Activity Score (BVAS) of 0 on a maximum prednisone dose of 4 mg/day [9, 10]. However, relapsing or refractory sinonasal manifestations due to comorbid CRS can occur during OCS tapering in the maintenance phase, which is an obstacle to achieving OCS-free remission in EGPA [11]. The comorbid CRS in EGPA is defined as the representative phenotype of a secondary diffuse CRS. However, its endotype dominance remains undefined. The comorbid CRS can be observed during the maintenance phase of EGPA [11], but the clinical features remain unclear.

This study aimed to retrospectively investigate the clinical features of comorbid CRS during the EGPA maintenance phase.

## Materials and methods

The present study was conducted at a single institution. The Research Ethics Committee of our institution approved the study protocol. Data were retrospectively obtained from patients with EGPA between August 1, 2021, and May 31, 2024. The opt-out method was used to obtain informed consent. All patients were over 18 years of age. Patients who lacked sinonasal histology data were excluded. 

At the first consultation with an otolaryngologist, all patients with EGPA presented with sinonasal symptoms such as nasal obstruction and postnasal drip, and were subsequently diagnosed with comorbid CRS based on sinonasal computed tomography (CT) findings. EGPA was diagnosed by rheumatologists following two guidelines [12,13]. To determine the phenotype of comorbid CRS, the EPOS 2020 criteria were used. When showing a histopathological eosinophilic count ≥10/ high-power field (HPF), according to the primary eCRS criteria defined by EPOS 2020 [1], patients with comorbid CRS were considered to have eosinophilic inflammation. Blood eosinophilia was defined as an absolute eosinophil count (AEC) of ≥500/μL.

No patients had been diagnosed with the primary eCRS at their first consultation with otolaryngologists. As collaboration between otolaryngologists and rheumatologists began, all patients had already received EGPA treatments: inhaled corticosteroid (ICS) for asthma control, and nasal corticosteroid (CS) spray for allergic rhinitis control; immunosuppressants (IS), MPZ, and OCS for EGPA control. At the time of the otolaryngology consultation, none of the patients showed signs of systemic relapse. The adjustment of OCS (prednisolone) dosages, the administration of 300 mg of MPZ every four weeks, and additional administration of IS, such as cyclophosphamide (13.3-15.0 mg/kg) and/or azathioprine (25-50 mg/body), were all performed by rheumatologists. 

The following clinical data were collected: age, sex, duration from the first consultation with otolaryngologists to the diagnosis of EGPA or the application of MPZ, body mass index (BMI), Brinkman index indicating smoking intensity, presence of allergic rhinitis and asthma, presence of myeloperoxidase anti-neutrophil cytoplasmic antibody (MPO-ANCA), Lund-Mackay CT total scores and its subdomain scores [14], BVAS at the time of EGPA diagnosis or at the first consultation with otolaryngologists during the maintenance phase of EGPA, presence of NPs, medication status, the Sino-Nasal Outcome Test-22 (SNOT-22) total scores [15], blood test including class of specific IgE to 13 allergens, and histopathological data. To understand the severity of CRS-related quality of life (QOL), SNOT-22 total scores were stratified into mild, moderate, and severe categories [16]. In the histopathological data, eosinophilic count (cells/HPF), basement membrane thickness, the grade of hyperplasia of goblet cells, and the severity of subepithelial edema were assessed.

Biopsies were obtained from NPs in NP-positive cases and from the ethmoid mucosa in NP-negative cases [4]. Hematoxylin and eosin-stained sections were analyzed, and histopathological eosinophil counts were obtained by averaging the three densest HPF areas (×400 magnification) per sample. The grade of hyperplasia of goblet cells and the severity of subepithelial edema were assessed using the methodology reported in a previous study [17].

Quantitative variables are presented as medians with interquartile ranges. Spearman’s rank correlation coefficients were calculated to investigate the correlation between histopathological eosinophilic count and BVAS/SNOT-22 total scores. For sub-analyses, patients were subdivided into two groups: OCS and OCS/MPZ. In the comparison between them, Mann-Whitney U tests were applied for continuous or ordinal variables, while Fisher’s exact tests were applied for dichotomous or categorical variables. A p-value of 5% was considered statistically significant. For all statistical analyses, the R software version 3.6.1 (R Foundation for Statistical Computing) was applied.

## Results

A total of 11 patients were included in this analysis. Patient characteristics are presented in Tables 1, 2.

**Table 1:** Clinical backgrounds and its comparison between OCS and OCS+MPZ groups In statistical analyses, the comparison between OCS and OCS/MPZ groups is performed. All values are presented as median (interquartile range) or as numbers (n). Fisher’s exact tests are applied for analyses of categorical variables, while Mann-Whitney U tests for analyses of continuous variables. A p-value of 5% is considered statistically significant. BMI: body mass index; BVAS: Birmingham Vasculitis Activity; CRS: chronic rhinosinusitis; CS: corticosteroid; CT: computed tomography; EGPA: eosinophilic granulomatosis with polyangiitis; ICS: inhaled corticosteroid; IS: immunosuppressant; MPO-ANCA: myeloperoxidase anti-neutrophil cytoplasmic antibody; MPZ: mepolizumab; NP: nasal polyp; OCS: oral corticosteroid; SNOT-22: 22-Item Sino-Nasal Outcome Test.

			All patients n = 11		OCS n = 4	OCS+MPZ n = 7	p-value
Age (y)								60 (53, 68)		58 (49, 67)	60 (56, 68)	0.70
Sex, male (n)			6		2	2	0.58
Duration (month)	From EGPA diagnosis		34 (17, 88)		94 (74, 136)	27 (16, 39)	0.11
	From MPZ application		-		-	18 (13, 33)	
BMI (kg/m^2^)			21 (20, 24)		20 (19. 21)	23 (20. 26)	0.32
Brinkman index			300 (0, 600)		300 (0, 625)	300 (0, 550)	1.00
Allergic rhinitis (n)			9		3	6	1.00
Current asthma (n)			6		2	4	1.00
MPO-ANCA-positive (n)			0		0	0	1.00
Lund-Mackay CT score	Total		10 (7, 17)		12 (7, 17)	10 (7, 17)	0.64
	Maxillary sinus		2 (0, 2)		1 (0, 2)	2 (1, 2)	0.61
	Anterior ethmoid sinus		4 (2, 4)		3 (2, 4)	4 (2, 4)	0.91
	Posterior ethmoid sinus			4 (2, 4)	4 (4, 4)	2 (2, 4)	0.30
	Frontal sinus		2 (0, 2)		1 (0, 2)	2 (1, 3)	0.36
	Sphenoid sinus		0 (0, 2)		0 (0, 1)	0 (0, 3)	0.51
	Ostiomeatal complex		2 (0, 4)		3 (2, 4)	0 (0, 4)	0.60
CRS with NP (n)			9		4	5	0.49
BVAS score	At EGPA diagnosis		4 (2, 5)		5 (4, 6)	2 (1, 4)	0.12
	At first consultation with otolaryngologists		4 (2, 5)		5 (4, 6)	2 (1, 4)	0.12
Treatment		ICS (n)	6		1	5	0.19
		IS (n)	6		1	5	0.19
		Nasal CS spray (n)	2		1	1	1.00
		OCS (mg/day)	4 (1, 5)		5 (5, 6)	1 (1, 4)	0.06
SNOT-22 total score			23 (15, 55)		21 (18, 24)	47 (11, 66)	0.78
Severity of SNOT-22 (n)	Mild		5		2	3	0.46
	Moderate		3		2	1	
	Severe		3		0	3	

**Table 2:** Findings obtained from blood test and sinonasal histology and its comparison between OCS and OCS+MPZ groups In statistical analyses, the comparison between OCS and OCS/MPZ groups is performed. All values are presented as the median (interquartile range). Mann-Whitney U tests are applied for statistical analyses. A p-value of 5% is considered statistically significant. AEC: absolute eosinophil count; CRP: C-reactive protein; DFA: Dermatophagoides farinae; HPF: high-power field; MPZ: mepolizumab; OCS: oral corticosteroid; WBC: white blood cell.

			All patients n = 11	OCS n = 4	OCS+MPZ n = 7	p-value	
Blood test	WBC (×10^3^/μl)							6.1 (5.3, 6.7)	6.3 (6.2, 6.7)	5.4 (5.2, 6.4)		0.23
	Neutrophils (×10^3^/μl)		3.7 (3.0, 4.5)	4.3 (3.7, 4.9)	3.5 (3.0, 4.1)		0.41
	Lymphocytes (×10^3^/μl)		1.3 (0.8, 2.0)	1.0 (0.9, 1.4)	1.8 (1.0, 2.0)		0.79
	AEC (/μl)		52 (29, 371)	397 (381, 523)	29 (27, 51)		0.01
	Basophils (/μl)		29 (22, 35)	49 (36, 61)	24 (20, 29)		0.01
	Monocytes (/μl)		416 (99, 828)	904 (551, 1269)	118 (99, 652)		0.65
	CRP (mg/dl)		0.2 (0.0, 0.3)	0.2 (0.1, 0.8)	0.1 (0.0, 0.3)		0.50
	IgE (IU/ml)		624 (99, 828)	904 (551, 1269)	118 (99, 652)		0.32
Class of specific IgE	Paddy		0 (0, 0)	0 (0, 0)	0 (0, 2)		0.33
	Weed		0 (0, 0)	0 (0, 0)	0 (0, 1)		0.33
	Food		0 (0, 0)	0 (0, 1)	0 (0, 0)		0.78
	Epithelium		0 (0, 0)	0 (0, 0)	0 (0, 0)		0.58
	Mold		0 (0, 2)	1 (0, 2)	0 (0, 2)		0.74
	Alder		0 (0, 2)	0 (0, 1)	2 (0, 2)		0.38
	Birch		0 (0, 2)	0 (0, 1)	0 (0, 2)		0.65
	Japanese cedar		0 (0, 3)	0 (0, 0)	2 (0, 4)		0.10
	Japanese cypress		0 (0, 0)	0 (0, 0)	0 (0, 2)		0.32
	DFA		1 (0, 3)	1 (0, 2)	2 (0, 3)		0.69
	House dust		1 (0, 3)	1 (0, 2)	2 (0, 3)		0.74
	S. aureus enterotoxin	A	0 (0, 2)	0 (0, 1)	0 (0, 2)		0.78
		B	0 (0, 0)	0 (0, 1)	0 (0, 0)		0.78
Sinonasal histology	Eosinophilic count (cells/HPF)		136 (78, 184)	143 (123, 177)	86 (43, 184)		0.65
	Basement membrane thickness (μm)		13 (11, 16)	12 (11, 13)	14 (12, 19)		0.41
	Goblet cell hyperplasia		1 (1, 1)	2 (1, 2)	1 (1, 1)		0.11
	Subepithelial edema		1 (1, 2)	1 (1, 2)	1 (1, 1)		0.83

**Figure 1:**
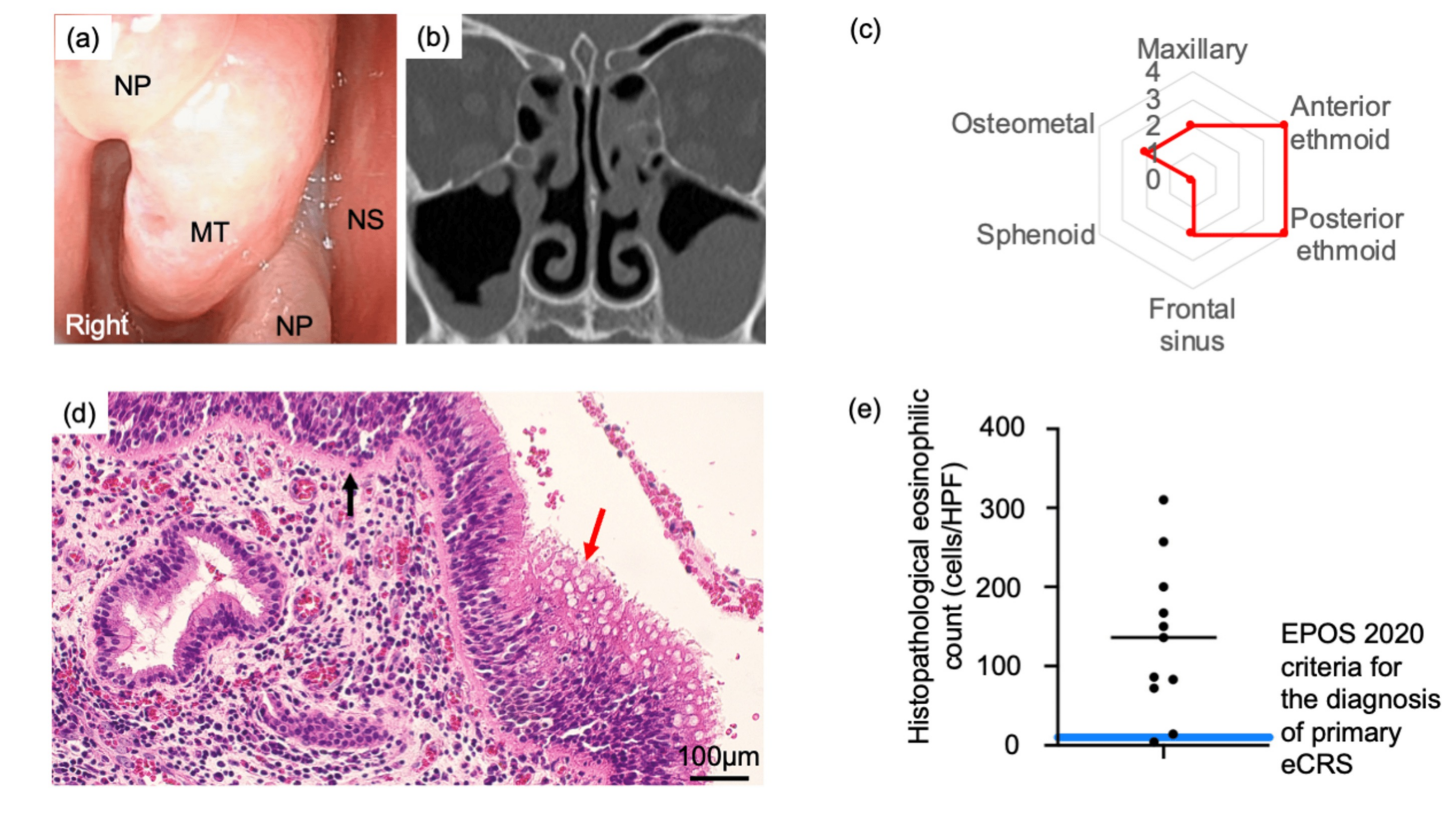
Endoscopic, sinonasal CT, and histopathological findings of comorbid CRS in the maintenance phase of EGPA (a) Representative endoscopic findings, (b) CT findings, (c) median values of each sinus lesion in the Lund-Mackay CT score, (d) sinonasal histological findings in the hematoxylin and eosin-stained section (black arrow: basement membrane; red arrow: subepithelial edema), and (e) distribution of histopathological eosinophilic count in sinonasal mucosa. A black line indicates the median value of the histopathological eosinophilic count, while a blue one the cut-off value for the primary eCRS defined by EPOS 2020. The findings in (a), (b), and (d) are obtained in a patient treated with OCS + MPZ. CT: computed tomography; eCRS: eosinophilic chronic rhinosinusitis; EGPA: eosinophilic granulomatosis polyangiitis; EPOS 2020: European Position Paper on Rhinosinusitis and Nasal Polyps; OCS: oral corticosteroid; MPZ: mepolizumab; MT: middle turbinate; NP: nasal polyp; NS: nasal septal

The median age, BMI, and Brinkman index (interquartile range, IQR) were 60 (53−68) years, 21 (20−24), and 300 (0−600), respectively. The median duration (IQR) from EGPA diagnosis to the first consultation with otolaryngologists was 34 (17−88) months. In patients treated with OCS+MPZ, the median duration (IQR) from the application of MPZ to the first consultation with otolaryngologists was 18 (13−33) months. The proportion of male patients was 55% (n=6). The median SNOT-22 total score (IQR) was 23 (15−55). In the SNOT-22 stratification, five (45%), three (27%), and three (27%) patients were classified into mild, moderate, and severe categories, respectively. Three patients had been positive for MPO-ANCA at the diagnosis of EGPA, while all patients were negative during the maintenance phase of EGPA. The median BVAS scores (IQR) at the time of EGPA diagnosis and at the first consultation with otolaryngologists were the same: four (2−5). At the first consultation with otolaryngologists, seven patients had a high BVAS score (≥4), which was mainly due to the persistent status of the nervous system (e.g., peripheral sensory neuropathy and motor multiple mononeuritis). In the other patients, the BVAS score of >0 was mainly due to ear, nose, and throat involvement. Nine (82%) and six (55%) patients had allergic rhinitis and asthma, respectively. Nine (82%) patients had NPs (Figure 1a). In the Lund-Mackay CT total score, the median value (IQR) was 10 (7−17). The main inflammation sites were the anterior and posterior ethmoid sinuses (Figures 1b, 1c). In AEC, the median value (IQR) was 52 (29−371)/μl (Table 2), and only one (9%) patient exhibited blood eosinophilia. Histopathological eosinophilic counts were determined by pathologists, and the median value (IQR) was 136 (78-184) cells/HPF (Figure 1d, Table 2). Ten (91%) patients met the criteria for primary eCRS (Figure 1e).

Six (55%), seven (64%), six (55%), and two (18%) patients received ICS, MPZ, IS, and nasal CS, respectively. All patients received OCS treatment, and the median value (IQR) of the OCS dose was 4 (1−5) mg/day. In correlation between histopathological eosinophilic count and BVAS at the first consultation with otolaryngologists, the correlation coefficient (p-value) was 0.51 (0.11). In correlation between histopathological eosinophilic count and SNOT-22 total score, the correlation coefficient (p-value) was -0.52 (0.11). No significant correlations were observed. 

For the sub-analyses, we compared the clinical features between the OCS and OCS+MPZ groups. Only two features showed significant differences: AEC and blood basophil count in the OCS+MPZ group were lower than those in the OCS group (Tables 1, 2). 

## Discussion

The current study demonstrated that the presence of NPs with eosinophilic inflammation can be observed under the maintenance therapy for EGPA and its comorbidities, in particular, even when performing MPZ treatment.

In the SNOT-22 stratification, one study targeting primary CRS reported that 15%, 95%, and 100% of patients felt that their QOL could be affected in mild, moderate, and severe categories, respectively [16]. In our study, 55% of the patients were classified into the moderate/severe category. Thus, patients can experience deterioration of CRS-related QOL during the maintenance phase of EGPA, in particular when tapering OCS dosages.

In the present study, over 80% of the patients had CRSwNPs, and the inflammation sites were mainly in the anterior and posterior ethmoid sinuses. The presence of NPs and the main inflammation sites of the ethmoid sinuses rather than the maxillary sinuses can be characteristic features of primary eCRS [4]. Therefore, the endoscopic and CT findings of comorbid CRS in the maintenance phase of EGPA might resemble those observed in the primary eCRS.

In AEC, over 90% of the patients did not show blood eosinophilia. At the onset of EGPA, the main clinical finding of AEC is hypereosinophilia (AEC ≥1500/μL) [8]. In our study, at the time of consultation with otolaryngologists, some patients experienced persistent disease manifestations related to peripheral sensory neuropathy and multiple motor mononeuritis, but none were in the active phase of EGPA. Therefore, various treatment modalities for EGPA and its comorbidities are effective in improving blood eosinophilia, resulting in the control of systemic relapse. In addition, the decrease in AEC might contribute to the decrease in eosinophil recruitment from blood to the sinonasal mucosa [18].

In the diagnosis of primary eCRS, the assessment of the histopathological eosinophilic count in the sinonasal mucosa is crucial. The comorbid CRS in EGPA is classified as the secondary CRS [1]. At present, there are no criteria for the histopathological eosinophilic count of sinonasal mucosa to define eCRS specific for the comorbid CRS. The current study referred to the EPOS 2020 criteria for primary eCRS, and over 90% of the patients met the criteria. These findings suggest that the histopathological eosinophil characteristics of comorbid CRS during the maintenance phase of EGPA may resemble those of primary eCRS. In the acute phase of EGPA, the comorbid CRS can be observed [9,19]. In the current study, the median duration from EGPA diagnosis (acute phase of EGPA) to the first consultation with otolaryngologists was 34 months. A comparison of the histopathological eosinophilic count between the acute and maintenance phases for each patient might contribute to the establishment of the cut-off values defining secondary eCRS, and a longitudinal study due to the collaboration between rheumatologists and otolaryngologists is needed. 

In the EPOS 2020 criteria, the endotype of comorbid CRS remains unclear [1]. The presence of NPs and the CT and histopathological eosinophil findings in the current study suggest that endotype dominance can be due to T2 cytokines. Therefore, the limited effectiveness of the current treatment for EGPA and its comorbidities, in particular MPZ treatment, on the control of sinonasal inflammation related to eosinophils, is noteworthy. A longitudinal study investigated the efficacy of MPZ on the eosinophil count of the sinonasal mucosa, AEC, and cytokine concentrations in patients with primary CRSwNPs [18]. The application of MPZ reduced the histopathological eosinophilic count (59%) and AEC (83%) and increased the concentration of some T2 cytokines (IL-4, IL-5, and IL-13) in NPs. The increase in T2 cytokines might be due to the local feedback loop of T2 cytokines derived from the functional absence of IL-5 and/or the decrease in sinonasal eosinophils. Our current study lacked information on cytokines in the sinonasal mucosa. Based on the concept of endotypic heterogeneity in primary CRS [7], endotypes of the comorbid CRS might also be heterogeneous. In addition to other T2 cytokines such as IL-4, T1/T3 cytokines may also play a crucial role in controlling residual eosinophilic inflammation. The approach focusing on the endotype of comorbid CRS can provide novel pathophysiology, affecting EGPA treatments.

**Figure 2:**
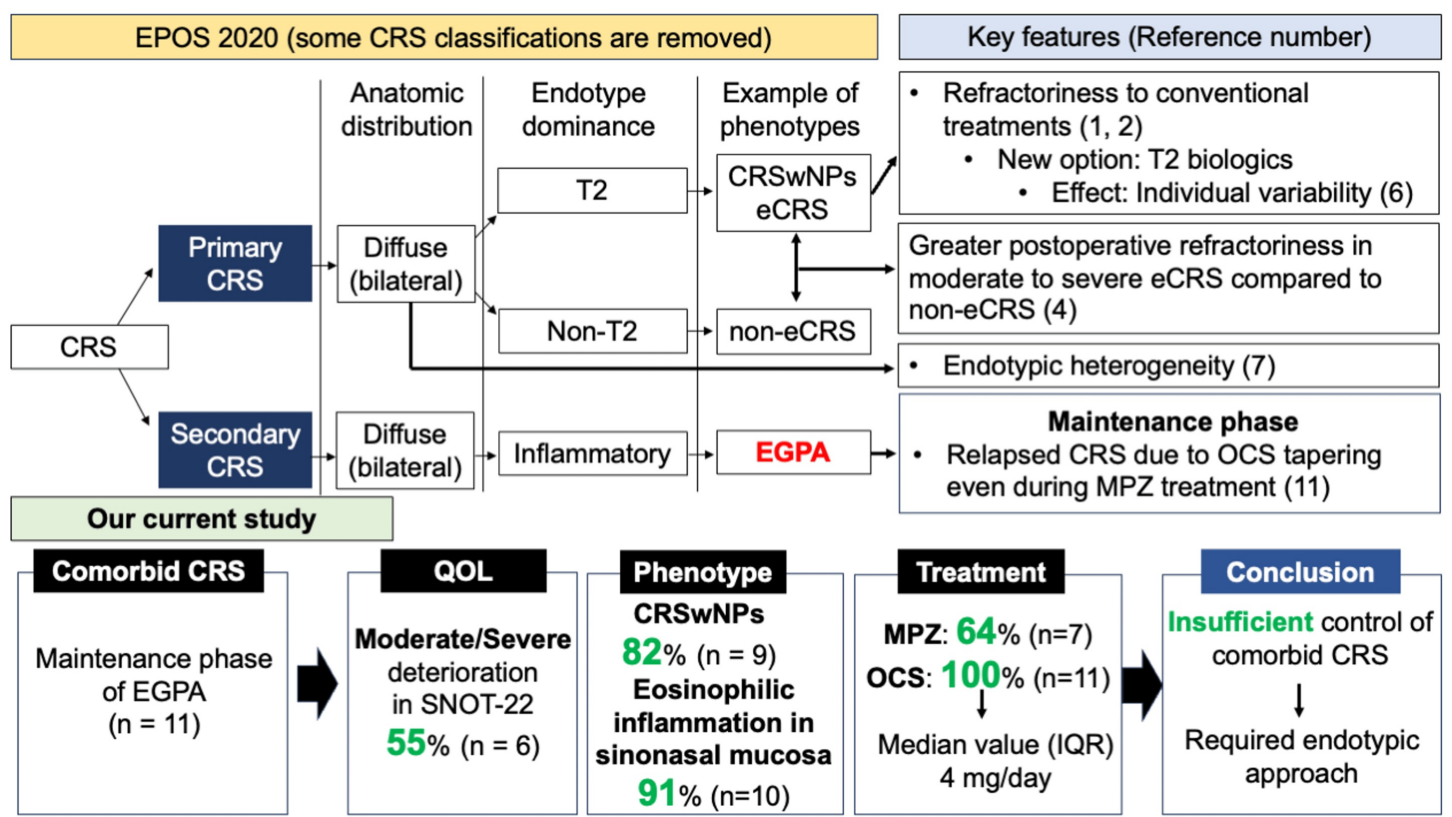
Classification of CRS in EPOS 2020 and findings observed in our current study. The image is created by the author. Our study focuses on the primary diffuse CRS and the comorbid CRS in EGPA. Therefore, other CRS phenotypes are excluded. CRSwNPs: chronic rhinosinusitis with nasal polyps; eCRS: eosinophilic chronic rhinosinusitis; EGPA: eosinophilic granulomatosis polyangiitis; EPOS: European Position Paper on Rhinosinusitis and Nasal Polyps; IQR: Interquartile range; OCS: oral corticosteroid; QOL: quality of life

A summary of the findings of the current study is shown in Figure 2. 

The current study had limitations. First, our findings were based on data from a single institution. As EGPA is a rare disease, the sample size was limited. Second, no patients had been diagnosed with primary eCRS before their first consultation with otolaryngologists. Therefore, it remains unclear whether these patients had primary eCRS prior to the acute phase of EGPA. Third, data were collected using a cross-sectional study design. To reveal the pathophysiology of comorbid CRS in more detail, a longitudinal study design focusing on not only sinonasal histology but also endotypic information using the sinonasal mucosa is needed.

## Conclusions

In conclusion, our study demonstrated that residual eosinophilic inflammation of the sinonasal mucosa can be observed during the maintenance phase of EGPA even after MPZ treatment. Further studies focusing on sinonasal endotypes during the maintenance phase of EGPA are required to elucidate the pathophysiology of comorbid CRS.
